# Assessment of Apple’s object capture photogrammetry API for rapidly creating research quality cultural heritage 3D models

**DOI:** 10.1371/journal.pone.0314560

**Published:** 2024-12-12

**Authors:** Stance Hurst, Lauren Franklin, Eileen Johnson

**Affiliations:** 1 Heritage and Museum Sciences, Museum of Texas Tech University, Lubbock, Texas, United States of America; 2 School of Anthropology, University of Arizona, Tucson, Arizona, United States of America; The University of Tulsa, UNITED STATES OF AMERICA

## Abstract

Photogrammetry is a significant tool museums utilize to produce high-quality 3D models for research and exhibit content. As advancements in computer hardware and software continue, it is crucial to assess the effectiveness of photogrammetry software in producing research-quality 3D models. This study evaluates the efficacy of Apple’s Object Capture photogrammetry API to create high-quality 3D models. The results indicate that Object Capture is a viable option to create research-quality models efficiently for a variety of natural and cultural heritage objects. Object Capture is notable for its minimal need for masking backgrounds within images and its ability to create models with fewer than 100 images and process 3D models in under 10 minutes.

## Introduction

In an effort to balance public engagement and research with the preservation of collections, museums actively are seeking ways to increase accessibility to their holdings [1–4]. 3D virtual replicas of objects increasingly are becoming recognized as a valuable tool for achieving this goal, allowing visitors and researchers to view museum objects. Virtual replicas are shared easily, maximizing research potential and increasing opportunities for those unable to access the materials in person. Additionally, these 3D models serve as a valuable resource for the conservation and documentation of artifacts and collections, effectively preserving a visual 3D form of them forever [5, 6].

Photogrammetry is the process of converting overlapping images into surface 3D data of objects and landscape features. Digital photogrammetry (i.e., structure-from-motion) has been recognized as a versatile and economical approach for creating 3D models of various objects in museum collections [7]. Investigators have used various photography techniques and software successfully to generate high-quality 3D models [3, 8–13]. One of the challenges in digitizing museum collections remains the significant amount of time and expertise required to produce high-quality 3D models.

Researchers are striving to identify effective workflows and software that can expedite and improve the accuracy of photogrammetric modeling [e.g., 6, 7, 11, 14, 15]. The relatively recent introduction of LiDAR technology in most smartphones and tablets has made 3D modeling even more accessible and user-friendly. Applications such as Scaniverse, PolyCam, and KIRI Engine allow users to combine laser guided measurements with photogrammetry texturization to generate fast, low resolution models of objects and landscape features [16]. LiDAR applications, however, typically do not offer much user control in the development process and generally are intended for devices that allow fewer photography settings (e.g., aperture, shutter speed, ISO, EV) to be changed.

Traditional photogrammetry computer software programs such as Agisoft Metashape, RealityCapture, and Bentley’s iTwin Capture Modeler tend to offer more editing workflow options. According to Kingsland’s [6] analysis of commonly used photogrammetry software among researchers, Metashape offers the highest level of control in all stages of 3D model development but also demands the longest computer processing time. On the other hand, RealityCapture requires the shortest processing time but lacks the same level of manual control and mesh quality/accuracy as Metashape. Metashape has the highest image alignment accuracy for objects specifically, although its texture resolution is not always higher when compared to other competing software [17]. iTwin Capture Modeler processing time sits between Metashape and RealityCapture. While it can produce sharper, higher resolution texture when compared to either, it cannot process 360 data and requires users to calibrate images manually by inputting camera lens and sensor information [18].

Assessing the cost of photogrammetry software and system requirements is essential when selecting the most suitable tool for museum-related projects. Agisoft Metashape’s software is the only one that is available for use for both Windows and MacOS operating systems and charges $549 for the educational version for non-commercial use. Epic Games/RealityCapture offers a complimentary Windows version for small businesses and educational institutions as of April 2024. Similarly, iTwin Capture Modeler has a free Windows version available for non-commercial use. Although free and open-source options like Meshroom and Regard 3D are available, these programs generally are more limited in processing capacity, scalability, and mesh quality compared to their higher-end counterparts.

Medina et al. [14] have demonstrated a photogrammetry workflow for creating 3D models of natural history collections. Using standardized imaging equipment and techniques, they have employed Reality Capture photogrammetry software successfully to produce accurate 3D models of natural history objects in 1–2 hours per object. Medina et al. [14] used this workflow to produce 1,000 3D models of natural history objects within a year [14].

Expanding upon that research, this paper introduces a novel workflow that utilizes Apple’s Object Capture photogrammetry API to produce expeditiously and efficiently research-quality 3D models of cultural and natural heritage. High-quality research 3D models are characterized by dimensional accuracy within 1mm and sufficiently high color and resolution to capture all object details. This research aims to assess Object Capture’s efficacy in generating 3D models using diverse cultural and natural heritage objects and employing various cameras and imaging techniques.

The diverse array of items reflects active research across the fields of archaeology, paleontology, and ethnology, encompassing a broad spectrum of material culture and natural heritage. These items include architectural structures, such as historic dugouts; faunal remains from the Pleistocene era; lithic tools and projectile points; pottery; and ethnographic figurines, each varying widely in material and morphology. The objects range in scale from artifacts measured in millimeters to expansive landscape models covering over 500 square meters, captured through drone-based photogrammetry. This variation in size and complexity underscores the need for photogrammetric tools capable of accommodating diverse documentation strategies, ensuring accurate 3D modeling for detailed analysis and interpretation.

Moreover, the research quality and effectiveness of Object Capture to generate 3D models also were examined using an additional dataset created by the second author. This additional dataset contained images of 11 lithic hand axes from the Tabun Cave (Israel) collection housed at the University of Arizona [19]. The second author successfully made 3D models from these images using Metashape software. The first author used the images to produce 3D models of the handaxes through Object Capture. The precision and resolution of these models were assessed by comparing physical caliper measurements with virtual measurements and by looking at the difference in polygon count as a measure of resolution. Both authors conducted these measurements individually and later compared them in a double-blind format.

## Apple Object Capture API

Apple introduced a new photogrammetry software API called Object Capture at its worldwide developer conference in June 2021. The API was restricted to Macs equipped with M-series or Intel processors with a 4GB AMD GPU and 16GB of RAM. In 2023, Apple released a more limited version of the Object Capture API for mobile iPhone and iPad devices.

Object Capture offered five processing workflows that generate varying model resolutions and texture maps (Table 1). In this workflow, mesh models and associated texture maps were generated directly without camera alignment and point cloud generation steps.

**Table 1:** Photogrammetry processing workflows offered by Object Capture API.

Processing Type	Polygon Count	Texture Map Resolution	Texture Map Types
Preview	25,000	1024 X 1024	diffuse
Reduced	25,000	2048 X 2048	diffuse, normal, ambient, occulusion
Medium	100,000	4096 X 4096	
High	250,000	8k	diffuse, normal, ambient occulusion, roughness, displacement
Raw	Up to 30 million	Up to 16k	diffuse

Object Capture also allows users to enable automated object masking. With object masking enabled, the background surrounding the object is ignored while creating the 3D model. Object Capture also recognizes user-generated masks if the pixels are black (RGB: 0,0,0). These masks can be created in image editing software, such as Adobe Photoshop. Currently, Object Capture in Photocatch is limited to images in JPEG, PNG, or HEIC formats.

Apps that currently utilize Object Capture include Easy Photogrammetry, 3D Object Capture, PhotoCatch, and 3-D Photos Pro. These apps are available for download on the Apple App Store. Object Capture also is available within Apple’s Reality Composer Pro software. Photocatch is used in this research due to its active development, its more advanced 3D model viewing capabilities, and its ongoing enhancements in measuring and scaling tools. As these tools continue to evolve, they hold the potential to eliminate the need for additional applications like MeshLab for measuring and scaling.

## Methods

### Cameras and equipment

Three kinds of cameras were used for digital photography. Most images were captured with Nikon 7200 cameras. The ISO was set to 100, and the aperture and shutter speeds were modified based on the object type and changing light conditions as needed. Three types of lenses were used with the Nikon cameras: Nikkor 28mm *f*/1.8, Nikkor AF-S VR Micro-NIKKOR 105mm *f*/2.8, and AF-S FX Micro-NIKKOR 60mm f/2.8. An iPhone 13 Pro Max was used to photograph two of the objects. Finally, aerial images for 3D landscape modeling were captured using a DJI Zenmuse X5 camera mounted to a DJI Inspire 1 unmanned aerial vehicle (UAV).

**Fig 1:**
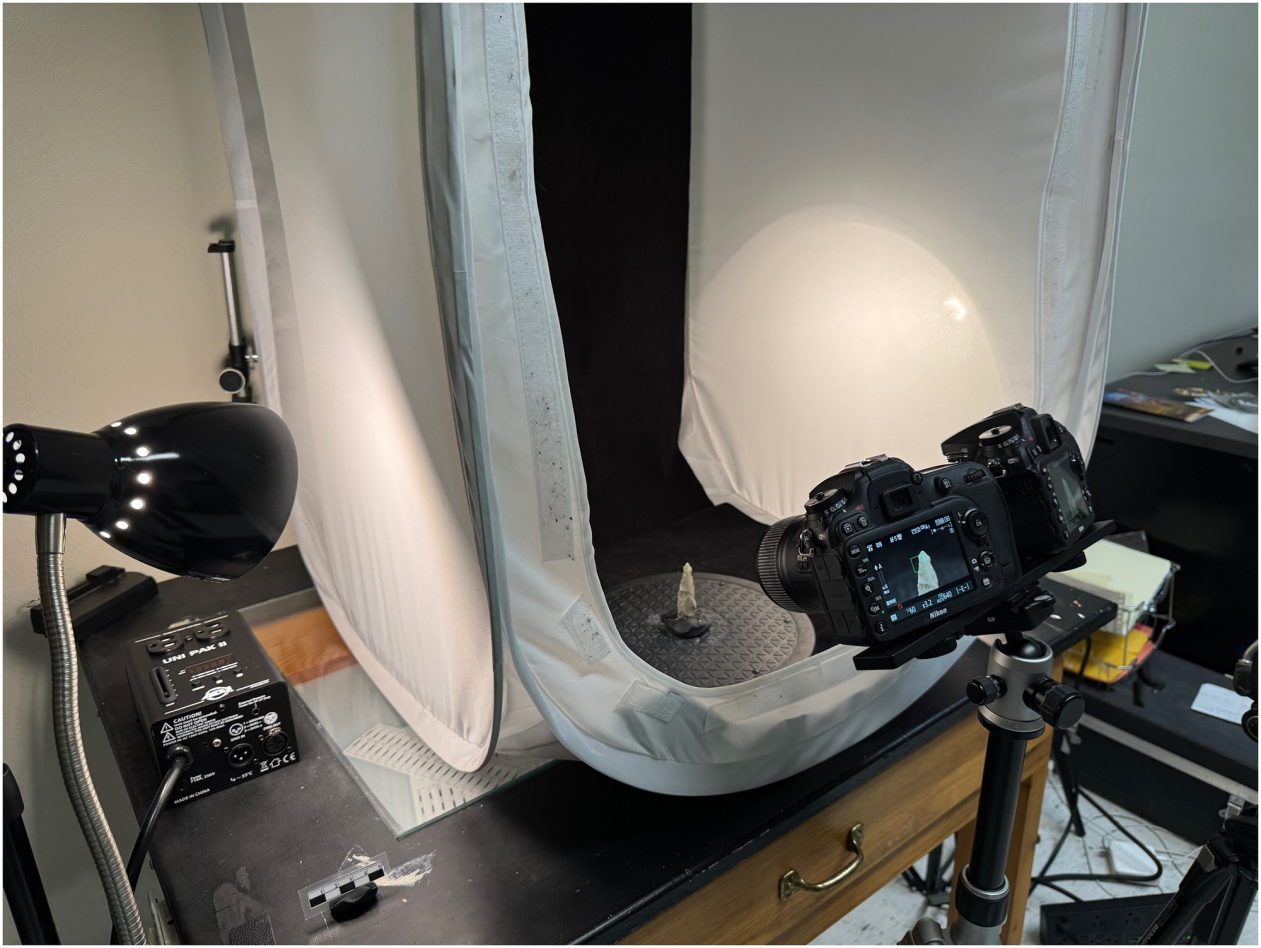
Photogrammetry Object Capture set up in the digital heritage lab at the Museum of Texas Tech University.

The equipment used for indoor object-based photogrammetry included tripods, a light tent, a lightbox, a turntable, and an X-Rite Photo ColorChecker Passport (Fig 1). To ensure the camera remained stable, exposures were controlled remotely using Smartshooter v4 software on a Macbook Pro laptop. The Smartshooter software also allowed for tethering multiple cameras, and in this case, it was used to pair two Nikon 7200s. Four desk lamps with 60-watt incandescent light bulbs, a light box with an integrated LED light system, or a ring LED light attached to the camera lens illuminated the objects. The objects were secured on a turntable with modeling clay and manually rotated 1 to 3 degrees to capture all angles. The objects also were rotated to capture the top and bottom sides. To standardize color, an X-Rite ColorChecker was used to create camera calibration profiles for different lighting conditions to maintain color accuracy.

No equipment was necessary for ground-based photogrammetry that involved capturing images outdoors of stationary objects or ground-level features [20]. To reduce shadows, only natural light was used in the late morning. The architectural feature was encircled several times at different distances to capture all angles.

A DJI Inspire 1 UAV was used for aerial-based photogrammetry. The drone was operated manually with a remote controller, and an iPad was attached to see the flight paths and take photos that overlap by 40–60%. The drone was used in the late morning to minimize the effect of shadows on the images.

### Image and 3D model processing

All images were shot in RAW format and imported into Adobe Lightroom for development. The images then underwent distortion, color aberration correction, and white balance adjustment based on the color profile created from the color chart. The color chart, however, was not used in ground or aerial-based photogrammetry. Finally, the images were converted into high-quality JPGs before being imported into Photocatch for 3D model creation.

The photogrammetry 3D models were processed on Apple Macbook Air or Pro laptops. The Macbook Air contained an M1 chip (8-core CPU; 7-core GPU) with 16 GB of RAM, while the Macbook Pro laptop was equipped with an M2 Max chip (12-core CPU; 30-core GPU) with 64 GB of RAM. Both laptops were updated to the Sonoma 14.0 operating system when creating the 3D models.

The 3D models were created with Photocatch v1.6 that utilizes the Object Capture API. They were generated using both raw and high-processing settings. Furthermore, object masking was enabled for all indoor object-based photogrammetry.

### Scaling and measurement

After the 3D models were created within Photocatch, they were exported as .obj (OBJ wavefront) 3D models for scaling within the open-source software Meshlab. Photocatch also offered the option to export 3D models in Universal Scene Description Zero (USDZ) or Polygon File Format (PLY) format. Other Object Capture software, such as Apple’s Reality Composer Pro, only allowed exporting the 3D models in USDZ format. Using the open-source software Blender, the USDZ format could be converted into other standard 3D file formats, such as OBJ.

Meshlab is robust software specifically designed for manipulating and refining 3D mesh models. Meshlab is the final step for accurately scaling 3D models in this workflow. While Photocatch and other Object Capture software offer some scaling capabilities, Meshlab’s measurement tools are more advanced.

Scaling objects in Meshlab involved identifying two distinct and easily measurable points on the 3D model that corresponded to specific locations on the physical object and could be measured using calipers. The scale ratio then was determined by dividing the virtual object’s measurement by the physical object’s measurement using a caliper. Once the model had been scaled, it was exported from Meshlab and saved as a scaled version of the 3D object.

### Accuracy and resolution of object capture—Evaluation

**Fig 2:**
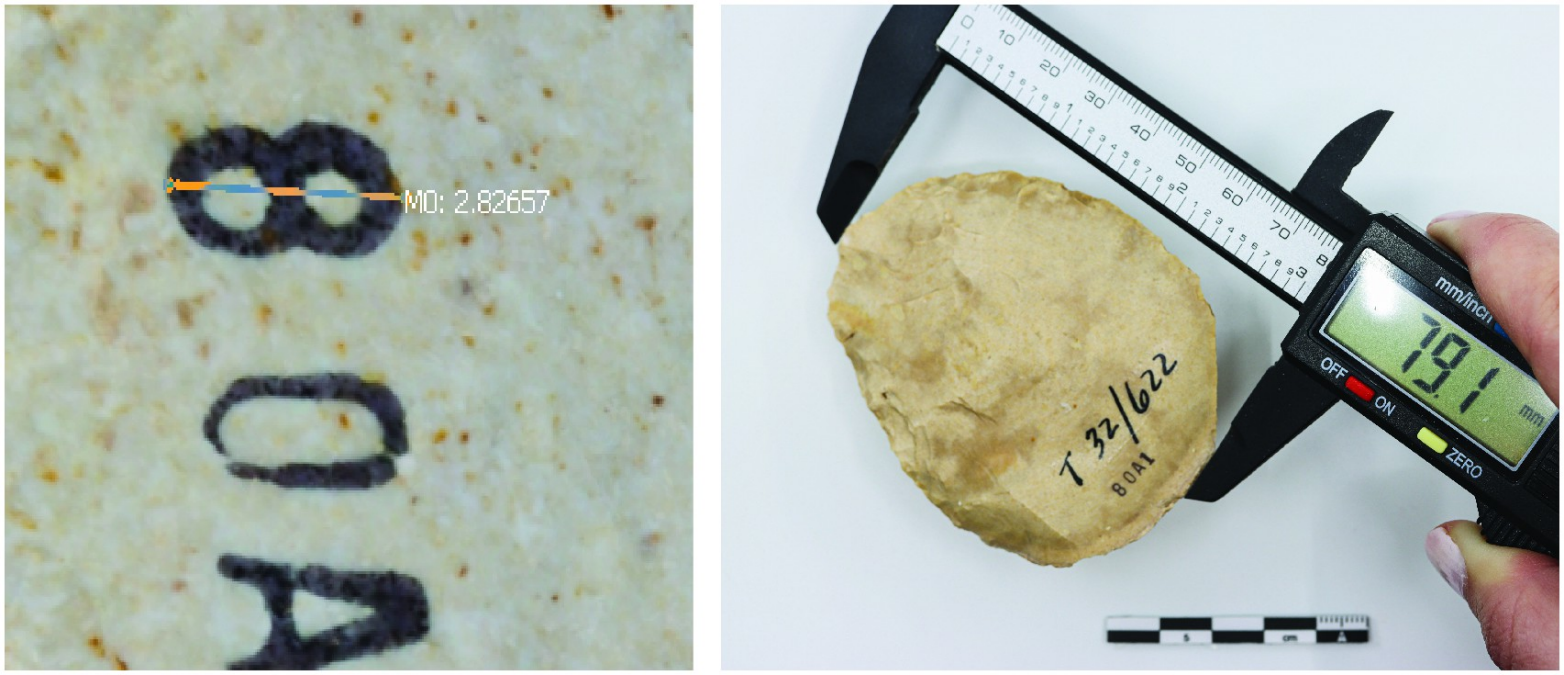
Measurements used to scale the 3D models in Meshlab: 2.8 mm based on a museum stamp and the total length of the object.

The handaxes from Tabun Cave were photographed with a Canon EOS 90D DSLR camera using a lightbox and Bluetooth turntable. Shots were taken of each individual hand axe half, sitting in a stand, from three different angles while the artifact turned. RAW images were directly placed into Metashape without any masking or processing. Metashape models were generated from depth maps to mesh without creating a dense/point cloud. Images were aligned with medium accuracy with a Key Point Limit of 40,000 and a Tie Point Limit of 10,000. Depth maps were generated with high quality to ensure no holes or missing elevation data. Depth maps were optimized by adjusting reconstruction uncertainty (10%), reprojection error (5%), and projection accuracy (5%). No masking was necessary. Finally, a texture was built over the mesh to represent the artifact surface accurately. Models were scaled based on small, identifiable museum stamp marks on the hand axes (Fig 2).

The first author used Agisoft Metashape 2.0 to scale, and the second author used Meshlab. When scaling the PhotoCatch models in Meshlab using the stamp marks, however, the results were less accurate than those in Metashape. To investigate this finding, alternate large-scale markers were selected: a point on each end of the total length of each handaxe. Both models were scaled accurately after using handaxe length instead of the museum stamp for scale. To ensure the accuracy of the Photocatch models, the total width of the hand axes measured in Meshlab was compared with physical caliper measurements of the actual objects, providing an independent validation of the model’s precision.

### Ethics statement

1a. The primary dataset (Table 1) is housed at the Museum of Texas Tech University in Lubbock, Texas, and the collection and analysis methodology complied with the terms and conditions for the source of the data.
1b. The secondary dataset from Tabun Cave, Israel (Table 4) is housed at the School of Anthropology, University of Arizona, Tucson Arizona, and the collection and analysis methodology complied with the terms and conditions for the source of the data.
1c. No human remain specimens were used in the study
1d. No permits were required for the described study, which complied with all relevant regulations.

## Results

Thirty-four 3D models have been generated in the primary dataset based on ongoing research at the Museum of Texas Tech University and the Lubbock Lake Landmark regional research program (Table 2). Most of these 3D models are viewable at the Museum’s Sketchfab website (https://sketchfab.com/MoTTU-heritage-lab). These models were created from a diverse set of objects, including seven bone artifacts, two ethnographic objects, a historic glass bead, and three metal objects from historical archaeology sites. Three landscape models were also captured, including a historical buffalo hunter’s dugout and a general landscape model captured with a UAV. The collection also includes 12 lithic objects comprising projectile points, a biface segment, a lithic core, five pieces of Casas Grande pottery, and a pipestem.

**Table 2:** Image capture settings for each of the 34 objects 3D modeled using Object Capture within Photocatch software.

No.	Object Description	Specimen No.	Material Type	Method	Total Images	Camera Model	Aperture and Shutter Speed	Focal Length	Masked?	Computer
1	Buffalo Hunter’s Dugout	n/a	Architectural Feature	ground-based	264	Nikon 7200	f11/3	28mm	No	M2
2	American Lion Phalanage	TTU-A1-220816	Bone	object-based	214	Nikon 7200	f8/1/4	28mm	Yes	M2
3	American Lion Phalange	TTU-A1-220817	Bone	object-based	166	Nikon 7200	f8/1/4	28mm	Yes	M2
4	Coyote Skull Comparative Collection	LLP-OC-591	Bone	object-based	98	Nikon 7200	f11/.6	28mm	No	M2
5	Ground Sloth Humerus	OAS-Crull-2	Bone	object-based	143	Nikon 7200	f13/3	28mm	No	M2
6	Ground Sloth Phalange	OAS-Wall-1	Bone	object-based	148	Nikon 7200	f11/1.6	28mm	No	M2
7	Ground Sloth Phalange	OAS-Crull-1	Bone	object-based	126	Nikon 7200	f11/1.6	28mm	Yes	M2
8	Macy Locality 100 Camel Skull	TTU-A1-200840	Bone	object-based	1189	Nikon 7200	f8/1/4	28mm	No	M2
9	Ethnographic Pottery Figurine	E3738	Ethnology Pottery Figurine	object-based	202	Nikon 7200	f13; 2.5	28mm	No	M2
10	Ethnographic Carved Wood Figurine	E3336	Ethnology Wood Figurine	object-based	274	Nikon 7200	f13/1.3	28mm	No	M2
11	Glass Bead	TTU-A1-260182	Glass	object-based	127	Nikon 7200	f11/1.3	105mm	Yes	M2
12	.50 Bullet from 4JK Locality 13	TTU-A1-276291	Historical Metal	object-based	110	Nikon 7200	f11/1/8	28mm	No	M2
13	4JK Locality 5 Eagle Button	TTU-A1-266254	Historical Metal	object-based	202	iPhone 13 Pro Max	f1.8/1/30	4.25mm	No	M2
14	Historical Pocket Knife	TTU-A1-256369	Historical Metal	object-based	160	Nikon 7200	f11/3	28mm	No	M2
15	Landscape Salt Creek	n/a	Landscape	aerial-based	519	DJI Zenmuse X5	f1.7/1/8	15mm	No	M1
16	Landscape Salt Creek	n/a	Landscape	aerial-based	519	DJI Zenmuse X5	f1.7/1/8	15mm	No	M1
17	Adair-Steadman Folsom Projectile Point	TTU-A7-45193	Lithic Material	object-based	71	Nikon 7200	f9/1/10	28mm	No	M2
18	Adair-Steadman Folsom Projectile Point	TTU-A7-45168	Lithic Material	object-based	64	Nikon 7200	f11/1/5	28mm	No	M2
19	Adair-Steadman Folsom Projectile Point	TTU-A7-45214	Lithic Material	object-based	68	Nikon 7200	f11/1/5	28mm	No	M2
20	Adair-Steadman Folsom Projectile Point	TTU-A7-45169	Lithic Material	object-based	86	Nikon 7200	f9/1/10	28mm	No	M2
21	Adair-Steadman Folsom Projectile Point	TTU-A7-45164	Lithic Material	object-based	89	Nikon 7200	f11/.76	60mm	No	M2
22	Adair-Steadman Folsom Projectile Point	TTU-A7-45234	Lithic Material	object-based	116	Nikon 7200	f13/.0625	60mm	No	M2
23	Adair-Steadman Folsom Projectile Point	TTU-A7-45174	Lithic Material	object-based	68	Nikon 7200	f13/.0625	60mm	No	M2
24	Adair-Steadman Folsom Projectile Point	TTU-A7-45176	Lithic Material	object-based	129	Nikon 7200	f13/.0625	60mm	No	M2
25	Caldwell Pedestal	TTU-A1-7080	Lithic Material	object-based	70	Nikon 7200	f11/.4	75mm	No	M2
26	Clovis Lithic Core	TTU-A5-3106	Lithic Material	object-based	122	Nikon 7200	f11/1	28mm	No	M1
27	Clovis Lithic Projectile Point	TTU-A7-60438	Lithic Material	object-based	89	Nikon 7200	f16/1/8	28mm	No	M2
28	Warnica-Wilson Plainview Lithic Projectile Point	TTU-A1-37121	Lithic Material	object-based	74	Nikon 7200	f14/3	28mm	Yes	M2
29	Casas Grande Pottery	TTU-A10-12	Pottery	object-based	133	Nikon DSLR 7200	f13/.6	28mm	No	M2
30	Casas Grande Pottery	TTU-A20-5	Pottery	object-based	83	Nikon DSLR 7200	f16/1	28mm	No	M2
31	Casas Grande Pottery	TTU-A10-5	Pottery	object-based	152	Nikon 7200	f13/.6	28mm	No	M2
32	Casas Grande Pottery	TTU-A3-55	Pottery	object-based	133	Nikon 7200	f13/.6	28mm	No	M2
33	Casas Grande Pottery	TTU-A6-88	Pottery	object-based	175	Nikon 7200	f13/2.5	28mm	No	M2
34	Clay Pipestem	TTU-A1-80	Pottery	object-based	123	Nikon 7200	f11/1	28mm	Yes	M2

**Fig 3:**
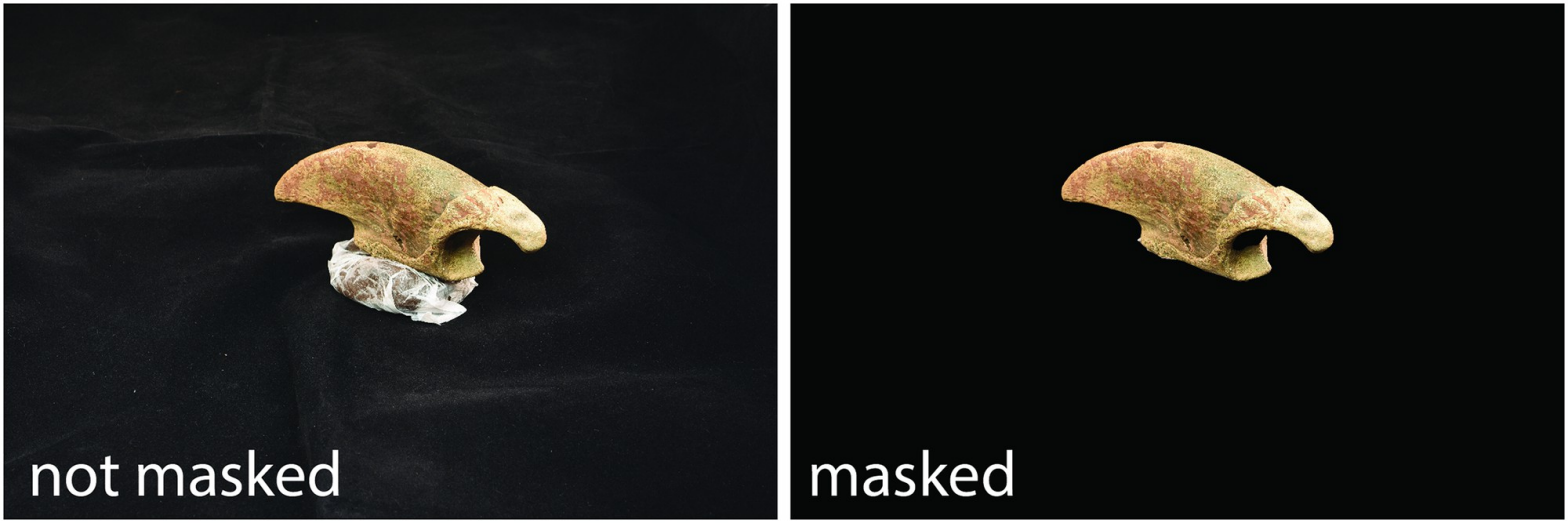
Ground sloth phalange (OAS-Crull-1) was masked in Adobe Photoshop to remove the cloth background and supporting modeling clay.

Image masking was needed for six (18%) objects (Table 2). Including a larger portion of the supportive clay base in these images necessitated masking as the clay moved with the object. As a result, a portion of the clay base was integrated into the 3D model, leading to distortion. To address this issue, the images were masked using the subject mask tool in Adobe Photoshop. This procedure effectively isolated the desired object. After selecting the object, the selection was inverted, and the background was eliminated, then filled in with black pixels (RGB: 0,0,0) (Fig 3).

**Fig 4:**
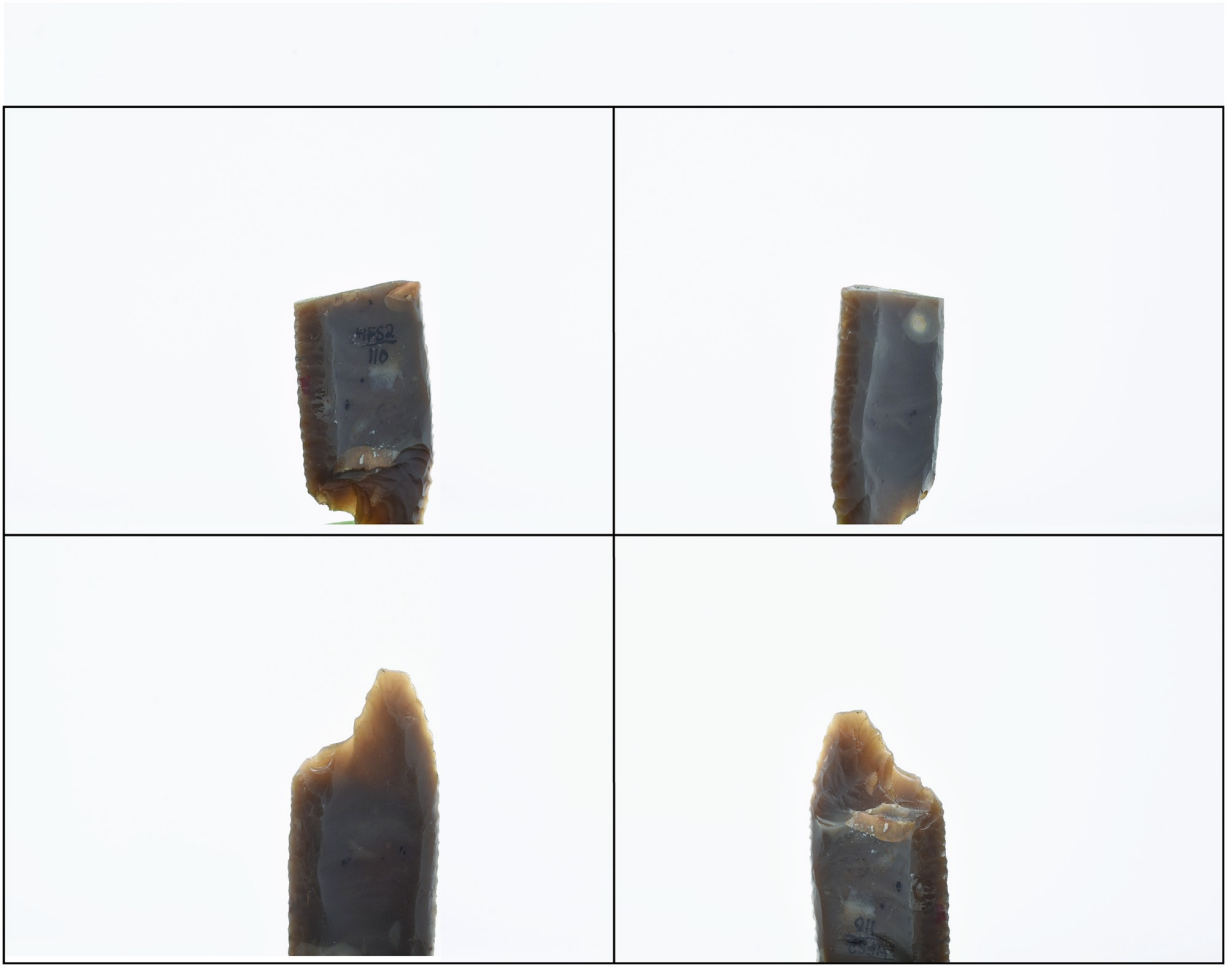
The Adair-Steadman Folsom projectile point (TTU-A7-45176) is framed partially during object-based photogrammetry to eliminate the need for image masking.

Masking can be avoided when using Object Capture if proper preparations are made before taking images. Most objects can be 3D modeled successfully without masking if the images do not show the supporting modeling clay. This method involves taking pictures of the objects with only a part of the object in each frame (Fig 4). After turning the object, the object is flipped, and the opposite part is captured in the frame. As a result, the images have overlapping parts of all objects from different angles, with the clay base being left out.

**Fig 5:**
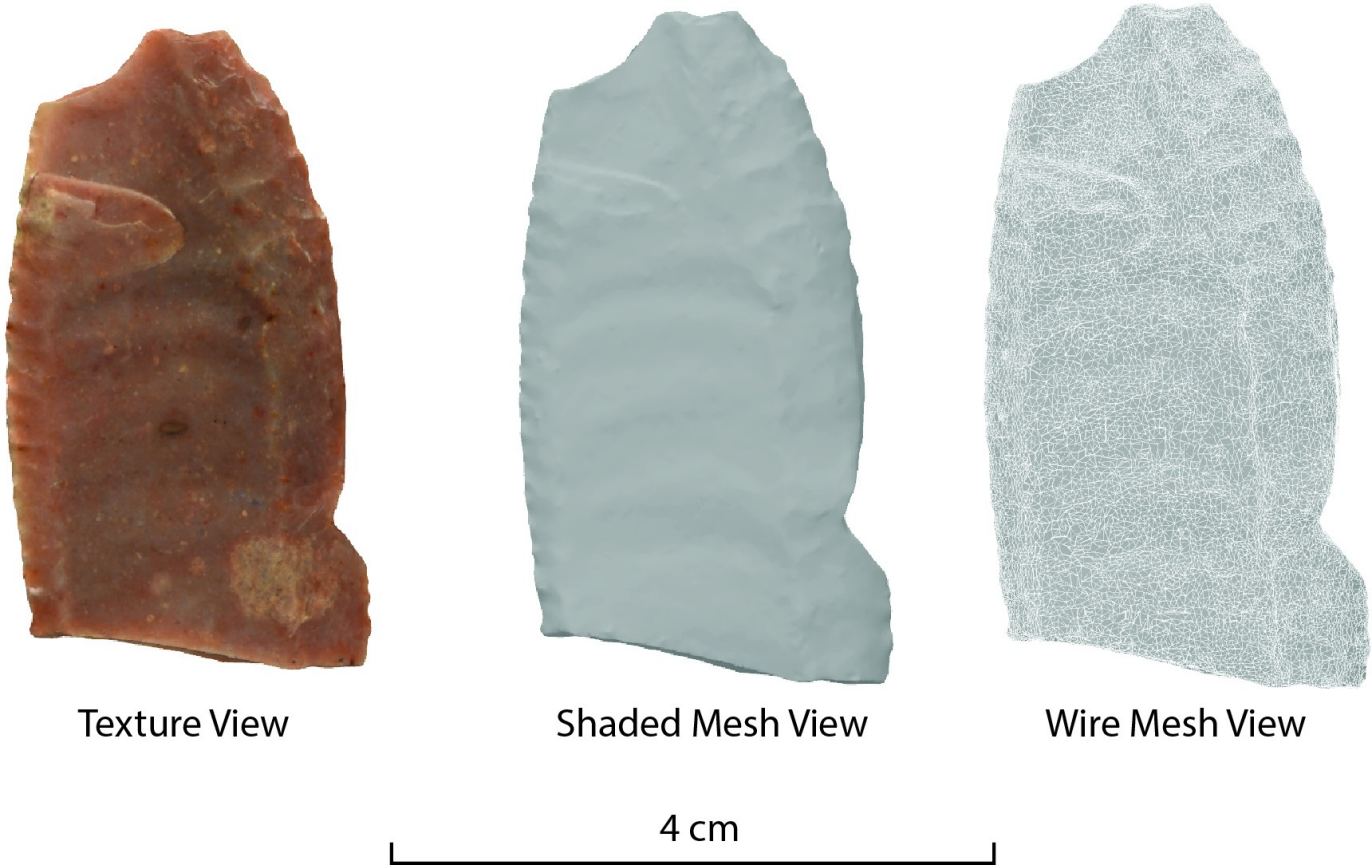
Folsom projectile point (TTU-A7-45169) from Adair-Steadman 3D modeled in Photocatch in 2 minutes and 10 seconds using 86 images.

3D modeling processing times in Photocatch ranged between 1 minute and 36 seconds to 1 hour and 49 seconds (Table 3). The notable difference in processing duration was ascribed to the substantial difference in the number of images acquired for each 3D model (from 64 to 1,189) and different shooting environments. Most (91%) of the 3D models were completed in less than 15 minutes, with 79.5% finished within 10 minutes. Also noteworthy is that 18 (53%) of the models were completed with only five minutes of processing time, and nine of these models required less than 100 images to generate a complete 3D model (Fig 5; Table 3). Over 250 images were processed for the 3D models that exceeded a 15-minute processing time, including the models that utilized aerial or ground-based photogrammetry techniques.

**Table 3:** Photogrammetry processing time and total polygon count for each 3D model created in Photocatch.

Number	Object Description	Specimen No.	Material Type	Total Images	Computer	Processing Type	Time (m = minutes s = seconds)	Total Polygon Count
1	Buffalo Hunter’s Dugout	n/a	Architectural Feature	264	M2	Raw	32m 27s	6,216,558
2	American Lion Phalanage	TTU-A1-220816	Bone	214	M2	Raw	4m 18s	101,860
3	American Lion Phalange	TTU-A1-220817	Bone	166	M2	Raw	3m 42s	37,062
4	Coyote Skull Comparative Collection	LLP-OC-591	Bone	98	M2	Raw	4m 6s	234,928
5	Ground Sloth Humerus	OAS-Crull-2	Bone	143	M2	Raw	13m 2s	630,642
6	Ground Sloth Phalange	OAS-Wall-1	Bone	148	M2	Raw	3m 38s	184,012
7	Ground Sloth Phalange	OAS-Crull-1	Bone	126	M2	Raw	8m 2s	201,860
8	Macy Locality 100 Camel Skull	TTU-A1-200840	Bone	1189	M2	Raw	44m 52s	404,883
9	Ethnographic Pottery Figurine	E3738	Ethnology Pottery Figurine	202	M2	Raw	8m 1s	346,078
10	Ethnographic Carved Wood Figurine	E3336	Ethnology Wood Figurine	274	M2	Raw	15m 31s	529,326
11	Glass Bead	TTU-A1-260182	Glass	127	M2	Raw	3m	69,506
12	.50 Bullet from 4JK Locality 13	TTU-A1-276291	Historical Metal	110	M2	Raw	3m 7s	36,368
13	4JK Eagle Button	TTU-A1-266254	Historical Metal	202	M2	Raw	7m 7s	95,566
14	Historical Pocket Knife	TTU-A1-256369	Historical Metal	160	M2	Raw	2m 11s	35,916
15	Landscape Salt Creek	n/a	Landscape	519	M1	High	57m 31s	249,998
16	Landscape Salt Creek	n/a	Landscape	519	M1	Raw	1h 1m	17,719,612
17	Adair-Steadman Folsom Projectile Point	TTU-A7-45193	Lithic Material	71	M2	Raw	1m 45s	49,304
18	Adair-Steadman Folsom Projectile Point	TTU-A7-45168	Lithic Material	64	M2	Raw	2m 3s	56,312
19	Adair-Steadman Folsom Projectile Point	TTU-A7-45214	Lithic Material	68	M2	Raw	2m 5s	30,708
20	Adair-Steadman Folsom Projectile Point	TTU-A7-45169	Lithic Material	86	M2	Raw	2m 10s	50,146
21	Adair-Steadman Folsom Projectile Point	TTU-A7-45164	Lithic Material	89	M2	Raw	2m 28s	209,014
22	Adair-Steadman Folsom Projectile Point	TTU-A7-45234	Lithic Material	116	M2	Raw	3m 19s	202,460
23	Adair-Steadman Folsom Projectile Point	TTU-A7-45174	Lithic Material	68	M2	Raw	4m 58s	143,966
24	Adair-Steadman Folsom Projectile Point	TTU-A7-45176	Lithic Material	129	M2	Raw	10m 7s	262,254
25	Caldwell Pedestal	TTU-A1-7080	Lithic Material	70	M2	Raw	4m 4s	387,000
26	Clovis Lithic Core	TTU-A5-3106	Lithic Material	122	M1	Raw	6m 52s	174,260
27	Clovis Lithic Projectile Point	TTU-A7-60438	Lithic Material	89	M2	Raw	7m 53s	515,044
28	Warnica-Wilson Plainview Lithic Projectile Point	TTU-A1-37121	Lithic Material	74	M2	Raw	2m 27s	118,858
29	Casas Grande Pottery	TTU-A10-12	Pottery	133	M2	Raw	1m 36s	32,650
30	Casas Grande Pottery	TTU-A20-5	Pottery	83	M2	Raw	6m 31s	631,924
31	Casas Grande Pottery	TTU-A10-5	Pottery	152	M2	Raw	7m 32s	320,102
32	Casas Grande Pottery	TTU-A3-55	Pottery	133	M2	Raw	8m 47s	665,470
33	Casas Grande Pottery	TTU-A6-88	Pottery	175	M2	Raw	10m 9s	653,714
34	Clay Pipestem	TTU-A1-80	Pottery	123	M2	Raw	14m 13s	665,472

### Object capture accuracy and resolution

The dimensional accuracy and resolution of 3D models made with Object Capture were assessed using the secondary dataset of 11 lithic hand axes. A distance of 2.8 mm between identifiable marks was used first as a scale. After scaling, the total width and length of the lithic hand axes were measured physically with a caliper and virtually within Meshlab. The average discrepancy in the width measurement between the physical and virtual methods was 1.48 mm (Table 4). The average discrepancy in the length measurement between the physical and virtual methods was 1.9 mm.

**Table 4:** The dimensional accuracy of Object Capture virtual 3D models measured in Meshlab compared to physical caliper measurements. The measurement difference was compared using a 2.8 mm museum stamp scale to the total object length.

Tabun Hand Axe (Cat No.)	Scale: Museum Stamp (2.8 mm)		Scale: Total Length	
	Total Width (mm)	Total Length (mm)	Total Width (mm)	Total Length (mm)
	Caliper—Meshlab Virtual Measure	Caliper—Meshlab Virtual Measure	Caliper—Meshlab Virtual Measure	Caliper—Meshlab Virtual Measure
T37-352	0.44	2.57	0.02	0.04
T30-1173	1.51	1.92	0.13	0.06
T37-374	2.14	1.01	0.84	0.91
T36-496	2.06	3.24	0.54	0.45
T38-348	2.61	4.52	0.37	0.08
T37-364	1.93	0.33	1.68	0.12
T32-622	0.39	1.18	0.60	0.21
T38-342	0.80	1.90	0.30	0.35
T44-35	1.56	1.25	0.07	0.05
T33-940	1.94	2.95	0.36	0.20
T33-988	0.93	0.01	0.26	0.11
Mean	1.48	1.90	0.47	0.23

Next, the total length of the objects was used as the scale to examine if it affected the dimensional accuracy of the 3D models. Using the total length of the object resulted in a difference of 0.23 mm between the virtual length measurement in Meshlab and the physical caliper measurement (Table 4). To verify accuracy, width was used as an independent measurement, revealing a difference of 0.47 mm between the physical caliper measurement and the virtual measurement in MeshLab (Table 4).

The Metashape 3D models had a higher polygon count than the Object Capture 3D models (Table 5). The mean difference in polygons per object between the Metashape 3D models and the Object Capture 3D models was more than a million. The most significant disparity in the number of polygons was 4,863,430, and the most minor disparity was 46,393 polygons.

**Table 5:** Comparison of mesh polygon counts between 3D models made using Photocatch (Object Capture) and Metashape.

Tabun Hand Axe (Cat No.)	Metashape Polygon Count	Object Capture Mesh Polygon Count*	Polygon Count Difference (Metashape–Object Capture Mesh)
30–1173	2,858,310	1,662,578	1,195,732
32–622	2,066,812	1,489,032	577,780
33–940	1,849,802	1,458,076	391,726
33–988	977,875	931,482	46,393
37–352	2,006,896	1,362,440	644,456
37–364	1,619,123	1,236,694	382,429
37–374	7,013,520	2,150,090	4,863,430
38–342	2,095,750	1,584,236	511,514
38–348	2,906,540	1,688,288	1,218,252
44–35	2,131,704	1,438,768	692,936
46–396	3,118,926	1,495,994	1,622,932
Mean difference			1,104,325.44

*Raw Processing type used in Photocatch

## Discussion

Using Apple’s Object Capture photogrammetry API within Photocatch software, various cultural and natural heritage objects and landscape elements were effectively 3D modeled. Moreover, Object Capture worked well with images from different shooting environments. For most object-based photogrammetry (Table 1), Object Capture’s automated background masking was effective, and no masking was required.

Image masking was needed for six (18%) of the objects. In these images, the clay support was visible that caused the 3D models’ misalignment. The manual creation of the masks in Adobe Photoshop for these images took 10 to 20 minutes per object. The masking time can be shortened by using Photoshop’s AI features to automate subject selection for masking the background. If the camera angles had been selected to hide the supporting materials from the images, these six objects could have been 3D modeled without masking.

**Fig 6:**
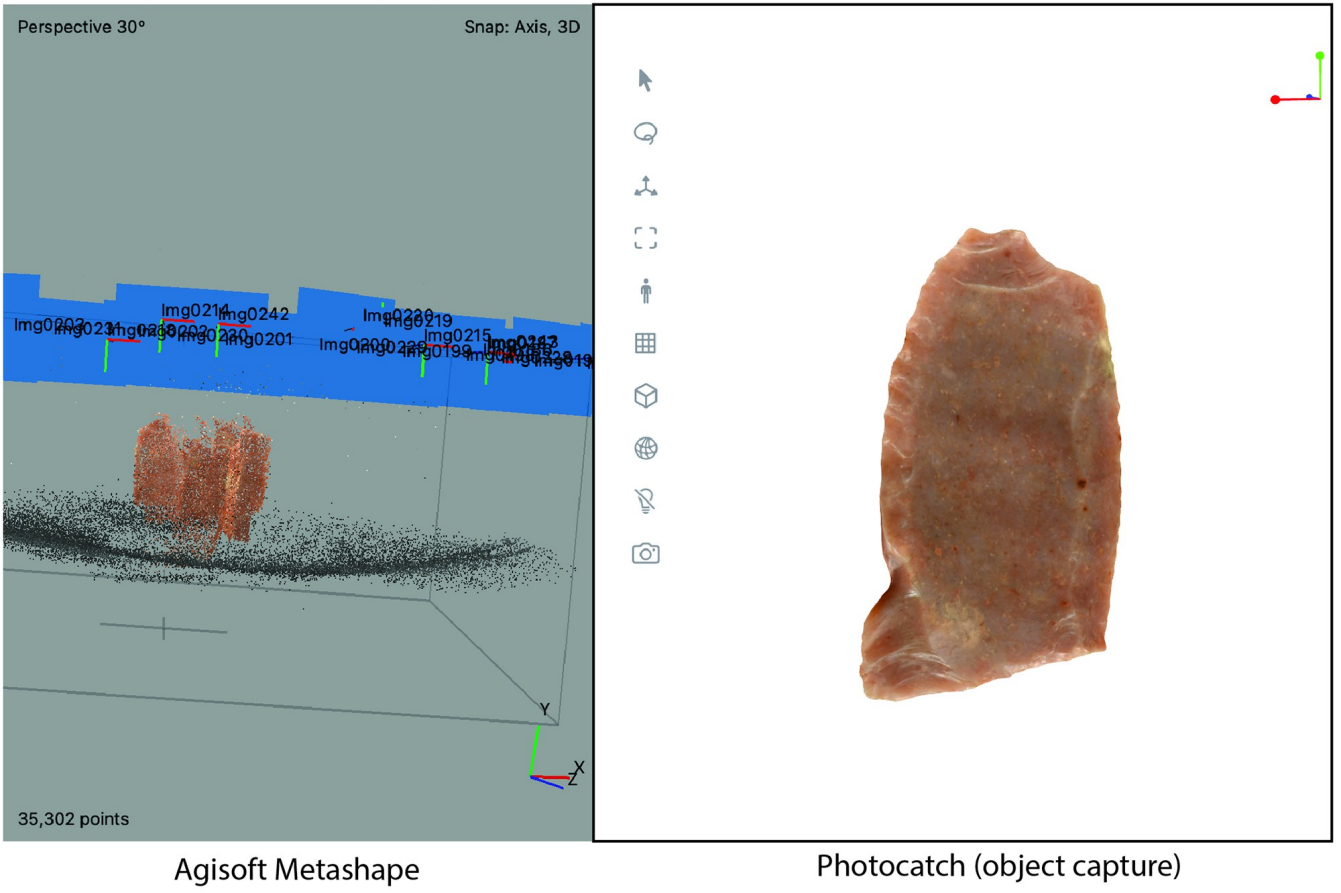
Comparison of Adair-Steadman Folsom projectile point (TTU-A7-45168) successfully aligned and 3D modeled in Photocatch to a failed alignment in Agisoft Metashape.

Many models successfully 3D modeled using Object Capture without masking may require additional images or masks using other photogrammetry software. To illustrate this point, the same images used to align two Adair-Steadman Folsom projectile points in Photocatch failed to align correctly using Metashape (Fig 6). Thin objects such as projectile points often require many more images or time-consuming and inefficient multiple-alignment strategies to create complete 3D models [11, 15].

The virtual models made by Object Capture were within 1 mm measurement accuracy compared to the physical objects. These results are comparable to those of other researchers [e.g., 21–23]. An important caveat is that developing a photogrammetry workflow with Object Capture requires other 3D modeling software and a scale encompassing most of the object.

When using a smaller scale based on an ink stamp, the first measurements of the 3D models of stone hand axes from Tabun Cave had more than a 1 mm discrepancy with the physical object (Table 4). The second author had created 3D models previously with Metashape software with less than 1 mm of dimensional error, using this smaller scale and two other small-scale measurements from the same stamp. The benefit of Metashape software was that it can use multiple measurements to scale the virtual object, unlike other 3D modeling software such as Meshlab that can only use one measurement for scaling. When using Object Capture to create a 3D model, therefore, it is important to have a scale that covers most of the object to minimize scaling errors. The second author used the ink stamp on the hand axes as a scale because the ink stamp had clear features that enabled a precise and repeatable measurement. Slight differences in the measurement of the smaller scale in Meshlab, however, created a more significant scaling error for the entire object. By contrast, the Metashape 3D models that used these smaller measurements for scaling were more accurate because the object could be scaled with multiple measurements.

**Fig 7:**
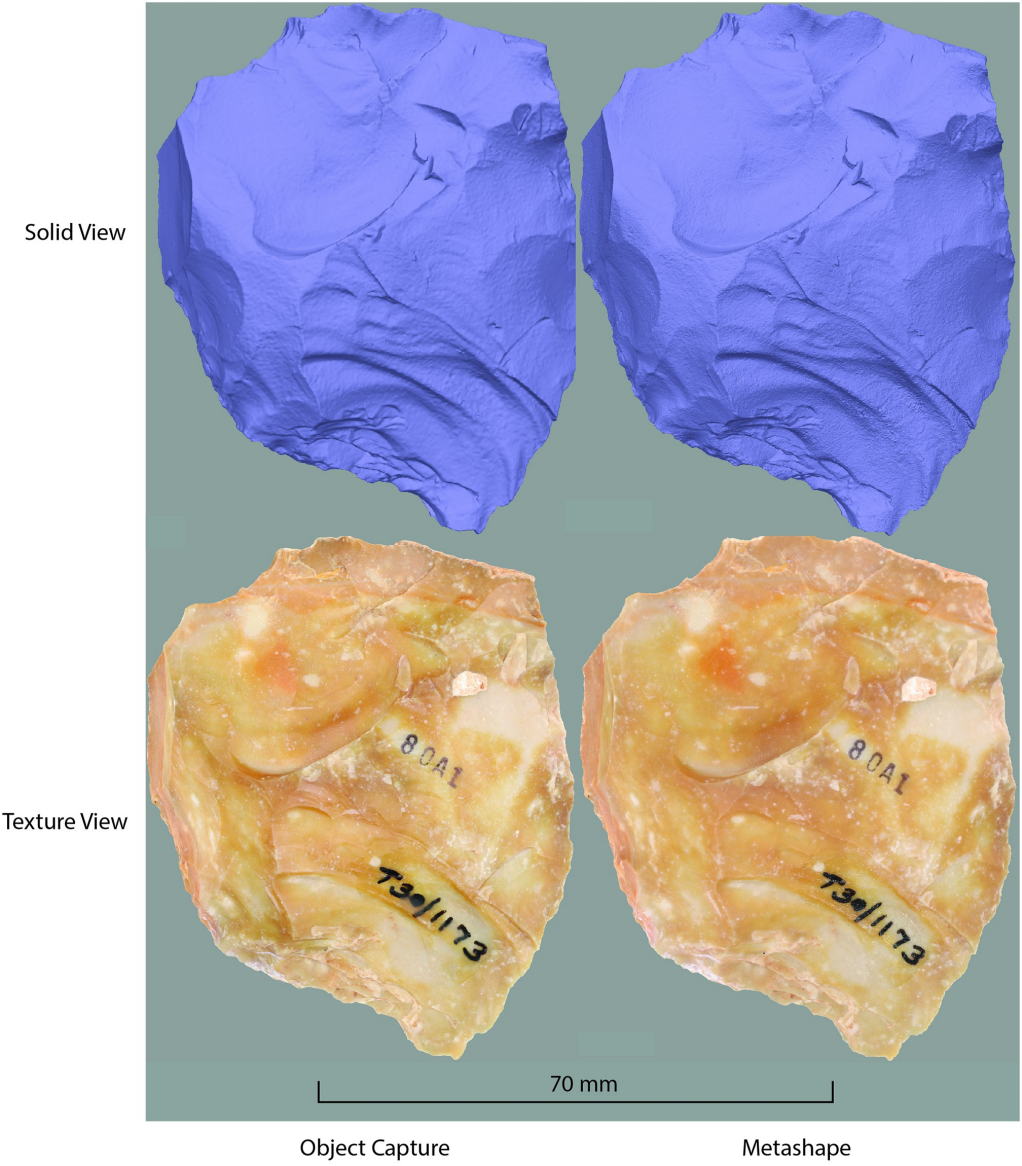
Comparison of the resolution between Metashape and Object Capture 3D models of a Tabun hand axe (30–1173). This hand axe exhibited the greatest discrepancy in polygon face counts (refer to Table 5). The fig was generated by importing the Object Capture OBJ file into Metashape. Screenshots of both the Object Capture and Metashape 3D models were taken, with the models scaled to the identical window size on a MacBook Pro laptop.

The 3D models created in Photocatch are highly detailed and showed small surface features, but the polygon face counts were lower than those produced by Metashape. From a research-oriented perspective, the lower polygon face counts of the Photocatch models are not prohibitive because they fulfilled the purpose of digitally viewing and comparing artifact collections or archaeological landscapes (Fig 7). Although the face counts are lower compared to those generated by Metashape, the models retain high resolution, ensuring that all object details remained clearly visible. This high level of visibility enables precise feature identification for analysis of the model’s elements. Further research is needed to determine the minimum polygon count required to produce a high-resolution 3D model suitable for research purposes, regardless of the photogrammetry software used.

Object Capture can create 3D models quickly with minimal expertise needed. Most models (91%) were finished within a 15-minute timeframe, with over half (53%) completed in under five minutes. These 3D modeling rendering times are faster than reported by most other researchers [6, 14].

Of particular note in this research is using fewer than 100 images to create research-quality object-based photogrammetry 3D models in less than five minutes. Using two tethered cameras in this photography setup, the images for these objects were captured within five minutes. With this Object Capture workflow, creating a photogrammetry 3D model from the start of image capture to a complete 3D model within 20 minutes is possible. The efficiency of 3D model creation depends on advancements in computer hardware and enhanced software photogrammetry algorithms. Photogrammetry software has improved significantly its speed and efficiency, now rivaling laser scanning in its ability to generate 3D models quickly [24–26].

## Conclusion

Apple’s Object Capture photogrammetry API rapidly and efficiently creates research-quality 3D models. Object Capture’s ability to work effectively across diverse cultural and natural heritage objects using a variety of cameras and imaging techniques is noteworthy. Most models are completed within a 15-minute time frame and require fewer than 100 images.

The results of this study have revealed specific challenges associated with using Object Capture. In certain instances, image masking is necessary. Other 3D modeling software and a scale encompassing most of the object must be considered when using this tool. In addition, Object Capture is available only to users with M-series Apple computers.

Despite these challenges, the study has shown that Object Capture can produce high-resolution models that maintain a high degree of measurement accuracy, even with lower polygon counts. This finding suggests that Object Capture has the potential to become an invaluable tool for heritage preservation and research by increasing accessibility to museum collections through the efficient creation of 3D virtual replicas of objects.

## References

[1] Magnani M, Guttorm A, Magnani N. Three-dimensional, community-based heritage management of indigenous museum collections: Archaeological ethnography, revitalization and repatriation at the Sámi Museum Siida. Journal of Cultural Heritage. 2018;31:162–9. doi: 10.1016/j.culher.2017.12.001

[2] Meier C, Berriel IS, Nava FP. Creation of a Virtual Museum for the Dissemination of 3D Models of Historical Clothing. Sustainability. 2021;13(22). doi: 10.3390/su132212581

[3] Ostrenga M. Photogrammetric Modeling of Museum Collections for Researcher Access [Masters thesis]. Lubbock: Texas Tech University; 2020.

[4] Wilson PF, Stott J, Warnett JM, Attridge A, Smith MP, Williams MA. Museum visitor preference for the physical properties of 3D printed replicas. Journal of Cultural Heritage. 2018;32:176–85. doi: 10.1016/j.culher.2018.02.002

[5] Angheluță LM, Rădvan R. Macro Photogrammetry for the Damage Assessment of Artwork Painted Surfaces. The International Archives of the Photogrammetry, Remote Sensing and Spatial Information Sciences. 2019;XLII-2/W15:101–7.

[6] Kingsland K. Comparative analysis of digital photogrammetry software for cultural heritage. Digital Applications in Archaeology and Cultural Heritage. 2020;18. doi: 10.1016/j.daach.2020.e00157

[7] Apollonio FI, Fantini F, Garagnani S, Gaiani M. A Photogrammetry-Based Workflow for the Accurate 3D Construction and Visualization of Museums Assets. Remote Sensing. 2021;13(3). doi: 10.3390/rs13030486

[8] Barba S, Barbarella M, Di Benedetto A, Fiani M, Gujski L, Limongiello M. Accuracy Assessment of 3D Photogrammetric Models from an Unmanned Aerial Vehicle. Drones. 2019;3(4). doi: 10.3390/drones3040079

[9] Evin A, Souter T, Hulme-Beaman A, Ameen C, Allen R, Viacava P, et al. The use of close-range photogrammetry in zooarchaeology: Creating accurate 3D models of wolf crania to study dog domestication. Journal of Archaeological Science: Reports. 2016;9:87–93. doi: 10.1016/j.jasrep.2016.06.028

[10] Lee M, Gerdau-Radonic K. Variation within physical and digital craniometrics. Forensic Sci Int. 2020;306:110092. doi: 10.1016/j.forsciint.2019.110092 31816484

[11] Magnani M, Douglass M, Porter ST. Closing the seams: resolving frequently encountered issues in photogrammetric modelling. Antiquity. 2016;90(354):1654–69. doi: 10.15184/aqy.2016.211

[12] Omari R, Hunt C, Coumbaros J, Chapman B. Virtual anthropology? Reliability of three-dimensional photogrammetry as a forensic anthropology measurement and documentation technique. Int J Legal Med. 2021;135(3):939–50. doi: 10.1007/s00414-020-02473-z 33244707

[13] Ozimek A, Ozimek P, Skabek K, Łabędź P. Digital Modelling and Accuracy Verification of a Complex Architectural Object Based on Photogrammetric Reconstruction. Buildings. 2021;11(5). doi: 10.3390/buildings11050206

[14] Medina JJ, Maley JM, Sannapareddy S, Medina NN, Gilman CM, McCormack JE. A rapid and cost-effective pipeline for digitization of museum specimens with 3D photogrammetry. PLoS One. 2020;15(8):e0236417. doi: 10.1371/journal.pone.0236417 32790700 PMC7425849

[15] Porter ST, Roussel M, Soressi M. A Simple Photogrammetry Rig for the Reliable Creation of 3D Artifact Models in the Field. Advances in Archaeological Practice. 2016;4(01):71–86. doi: 10.7183/2326-3768.4.1.71

[16] Robinson M. Photogrammetry for Archaeological Objects: A Manual. Sydney, Australia: Sydney University Press; 2024.

[17] Kingsland K. A Comparative Analysis of Two Commercial Digital Photogrammetry Software for Cultural Heritage Applications. New Trends in Image Analysis and Processing. In: Cristani M, Prati A, Lanz O, Messelodi S, Sebe N, editors. New Trends in Image Analysis and Processing–ICIAP 2019. 11808. Cham, Switzerland: Springer, Cham; 2019.

[18] Becker RE, Galyada LJ, MacLaughlin MM. Digital Photogrammetry Software Comparison for Rock Mass Characterization. 52nd US Rock Mechanics/Geomechanics Symposium; Seattle, Washington2018.

[19] Jelinek AJ. The Tabun Cave and Paleolithic Man in the Levant. Science. 1982;216:1369–75. doi: 10.1126/science.216.4553.1369 17798344

[20] Magnani M, Douglass M, Schroder W, Reeves J, Braun DR. The Digital Revolution to Come: Photogrammetry in Archaeological Practice. American Antiquity. 2020;85(4):737–60.

[21] Juckette C, Richards-Rissetto H, Aldana HEG, Martinez N. Using Virtual Reality and Photogrammetry to Enrich 3D Object Identity. 2018 3rd Digital Heritage International Congress (Digitalheritage) Held Jointly with 2018 24th International Conference on Virtual Systems & Multimedia (Vsmm 2018). 2018;164:406–10.

[22] Nuttens T, Maeyer PD, Wulf AD, Goossens R, Stal C, editors. Comparison of 3D accuracy of terrestrial laser scanning and digital photogrammetry: an archaeological case study. 31st EARSeL Symposium: Remote Sensing and Geoinformation Not Only for Scientific Cooperation; 2011; Prague, Czech Republic.

[23] Sapirstein P. Accurate measurement with photogrammetry at large sites. Journal of Archaeological Science. 2016;66:137–45. doi: 10.1016/j.jas.2016.01.002

[24] Armstrong BJ, Blackwood AF, Penzo-Kajewski P, Menter CG, Herries AIR. Terrestrial laser scanning and photogrammetry techniques for documenting fossil-bearing palaeokarst with an example from the Drimolen Palaeocave System, South Africa. Archaeological Prospection. 2017;25(1):45–58. doi: 10.1002/arp.1580

[25] Barszcz M, Montusiewicz J, Paśnikowska-Łukaszuk M, Sałamacha A. Comparative Analysis of Digital Models of Objects of Cultural Heritage Obtained by the “3D SLS” and “SfM” Methods. Applied Sciences. 2021;11(12). doi: 10.3390/app11125321

[26] Wrobel GD, Biggs JA, Hair AL. Digital Modeling for Bioarchaeologists. Advances in Archaeological Practice. 2019;7(1):47–54. doi: 10.1017/aap.2018.47

